# Supplementary material for: Does regional diversity recover after disturbance? A field experiment in constructed ponds
Source: PeerJ. 2016 Oct 18;4:e2455. doi: 10.7717/peerj.2455 (PMC5075687; doi:10.7717/peerj.2455)
Supplement: Table S1 — The fitted model included the fixed effects of rotenone treatment, sampling date, and the interaction between rotenone treatment and sampling date, as well as the random effect of pond identity. P-values <0.05 are indicated in bold. [file peerj-04-2455-s001.pdf]

**Variable**	***df***	**den *df***	***F***	***P***
**Species Richness**				
Rotenone	1	10	11.201	**< 0.0074**
Date	2	20	6.044	**< 0.0088**
Rotenone x Date	2	20	4.352	**< 0.0270**
**Chao1**				
Rotenone	1	10	8.901	**< 0.0137**
Date	2	20	0.735	< 0.4919
Rotenone x Date	2	20	5.081	**<0.0164**
