# Supplementary material for: Does regional diversity recover after disturbance? A field experiment in constructed ponds
Source: PeerJ. 2016 Oct 18;4:e2455. doi: 10.7717/peerj.2455 (PMC5075687; doi:10.7717/peerj.2455)
Supplement: Table S2 — Species can have a maximum occupancy of six ponds. The complete loss of a species from rotenone ponds is denoted by orange shading. The colonization or recolonization of a species is denoted by blue. [file peerj-04-2455-s002.pdf]

		Occupancy		
	Species	Pre Rotenone	1 Week Post	1 Year Post
Macroinvertebrates	Acilius fraternus	1	4	2
	Acilius mediatus	2	2	0
	Acilius spp larvae	3	2	3
	Agabus spp larvae	2	0	1
	Berosus sp.	0	0	1
	Chaoborus spp larvae	4	4	3
	Chauliodes spp larvae	2	0	2
	Chironomus spp larvae	4	5	4
	Coenagrionidae Family	1	0	2
	Desserobdella sp.	1	0	0
	Enochrus sp.	1	0	1
	Gyraulus parvus	1	0	2
	Helisoma anceps	1	0	1
	Helisoma trivolvis	1	1	1
	Helobdella sp.	2	0	1
	Hesperocorixa sp.	2	1	1
	Hydrobiomorpha sp.	1	1	1
	Hydrochara sp.	1	0	0
	Hydroporus sp.	0	0	1
	Laccophilus maculosus	4	4	4
	Laccophilus spp larvae	2	0	1
	Libellula luctuosa	0	0	2
	Libellula pulchella	0	0	1
	Musculium transversum	3	1	4
	Notonecta irrorata	4	1	1
	Oligochaeta	1	1	2
	Pachydiplax longipennis	4	0	3
	Physa gyrina	5	3	3
	Physella acuta	1	1	5
	Peltodytes litoralis	1	1	0
	Plathemis lydia	1	0	0
	Pseudosuccinea columella	0	0	1
	Sigara sp.	1	0	0
	Tropisternus spp larvae	0	0	2
Zooplankton	Acanthocyclops sp.	0	0	4
	Alona sp.	2	2	1
	Anuraeopsis sp.	1	2	3
	Asplanchna sp.	0	1	3
	Brachionus sp.	0	1	0
	Ceriodaphnia laticaudata	1	0	0
	Cerodaphnia lacustris	0	2	0
	Chydorus sp.	0	3	4
	Daphnia ambigua	2	0	0
	Daphnia longiremus	2	0	3
	Daphnia magna	2	2	0
	Daphnia pulex	1	0	0
	Diacyclops thomasi	0	0	2
	Diaphanosoma birgei	4	0	0
	Encentrum sp.	0	0	1
	Hexarthra mira	2	0	3
	Lecane inermis	0	0	1
	Lecane mira	4	3	4
	Lecane tenuiseta	0	2	0
	Lepadella acuminata	1	0	1
	Lophochari sp.	0	1	1
	Microcyclops rubellus	5	4	3
	Monostyla bulla	1	0	4
	Monostyla copeis	1	0	1
	Monostyla cornuta	1	1	3
	Monostyla quadridentata	0	0	1
	Mytillina ventralis	0	0	3
	Ostracoda	3	4	1
	Platyias patulus	1	0	0
	Rotifer sp A	1	1	2
	Rotifer sp B	0	1	1
	Rotifer sp C	1	1	1
	Scapholeberis mucronata	1	0	1
	Simocephalus vetulus	6	0	3
	Trichocerca sp.	1	0	0
	Tropocyclops sp.	0	0	3
