# Supplementary material for: Does regional diversity recover after disturbance? A field experiment in constructed ponds
Source: PeerJ. 2016 Oct 18;4:e2455. doi: 10.7717/peerj.2455 (PMC5075687; doi:10.7717/peerj.2455)
Supplement: File S2 [file peerj-04-2455-s004.rtf]

###Null Model for Extinction###Outputs the null model expectation for Gamma (regional richness) of a series of sites if extinction was a random process###Need to set working directory###Input files need to be set up with Species as Rows and Sites as Columns, saved as .txt., and names in “ ” when calling function###OutputFileName needs to be in " " when calling function and end with .csv###################################################################################################ExtNullModel = function(PreCommunityFileName, PostCommunityFileName, OutputFileName, numsims){###Data file needs to be set up with species as rows and sites as columnsCommunity_Pre = read.table(file = PreCommunityFileName, header=TRUE, row.names=1)Community_Post = read.table(file = PostCommunityFileName, header=TRUE, row.names=1)###Convert abundance matrices to incidence matricesCommunity_Pre = ifelse(Community_Pre >0, 1, 0)Community_Post = ifelse(Community_Post >0, 1, 0)###Determining site richness and species occupancy for pre and post sampling matricesRichness_pre = colSums(Community_Pre)Occupancy_pre = rowSums (Community_Pre)Richness_post = colSums (Community_Post)Occupancy_post = rowSums(Community_Post)nsites = ncol(Community_Pre)gamma = nrow(Community_Pre)ExpOcc = matrix(nrow = gamma, ncol = numsims)RandExtirp = matrix(nrow = gamma, ncol = nsites)###Counters in case a post site has more species than it did preMoreSpPost = 0SiteMoreSpPost = vector(mode = "numeric")SiteMoreSpCounter = 1###Null Model Simulationfor (i in 1:numsims){	for (j in 1:nsites)	{		###Identifying and counting the number of species pre disturbance		PreSite = Community_Pre[,j]		PreSiteWsp = which(PreSite>0)		PreNumSp = length(PreSiteWsp)				###Counting the number of species post disturbance		PostNumSp = Richness_post[j]		###Number of species lost from site		NumLost = PreNumSp - PostNumSp				###check to see if there are more species in a site post disturbance than pre disturbance		###If so, the simulation is not appropriate for the data and a message will print out at the end 		if(NumLost < 0)		{			RandExtirp[,j] = PreSite						if(i == 1)			{				MoreSpPost = 1				SiteMoreSpPost[SiteMoreSpCounter] = j				SiteMoreSpCounter = SiteMoreSpCounter +1			}		}			### If species richness did not change in a site between pre and post date then the site remains the same		if (NumLost == 0)		{			RandExtirp[,j] = PreSite		}		###If species richness was lost, then species are randomly lost from the pre site 		if (NumLost > 0)		{			RandomExt = sample(PreSiteWsp, NumLost, replace = FALSE, prob = NULL)						for(k in 1:NumLost)			{				PreSite[RandomExt[k]] = 0			}						RandExtirp[,j] = PreSite				}			}	ExpOcc[,i] = rowSums(RandExtirp, na.rm = FALSE, dims =1)}###Creating a place to store the gamma values from each simulationSimGammas = length(numsims)###Sum up the expected gamma for each simulationfor(l in 1:numsims){	SpPresent = which(ExpOcc[,l] > 0)	SimGammas[l] = length(SpPresent)}###Calculating 95% range values for the expected gamma from the simulationSimGammas = sort(SimGammas)mean = mean(SimGammas)lower95Range= SimGammas[round(numsims * .05)]upper95Range = SimGammas[round(numsims * .95)]###putting together simulation output that will be printed in the R consoleoutput = vector(length = 4)output[1] = meanoutput[2] = lower95Rangeoutput[3] = upper95Rangeoutput[4] = numsimslabels = vector(length = 4)labels = c("Mean Gamma", "Lower 95% Range", "Upper 95% Range", "Number of Simulations")###write an output file with all of the simulated gamma values write.csv(SimGammas, OutputFileName)###printing a note into the R console if model assumptions were violatedif(MoreSpPost >0){	print(c("More species post than pre for site", SiteMoreSpPost))}###printing a summary of the simulation in the R consoleFinal = cbind(labels, output)print(Final)}
